# Identification and characterization of the highly polymorphic locus D14S739 in the Han Chinese population

**DOI:** 10.3325/cmj.2015.56.482

**Published:** 2015-10

**Authors:** Chengchen Shao, Yaqi Zhang, Yueqin Zhou, Wei Zhu, Hongmei Xu, Zhiping Liu, Qiqun Tang, Yiwen Shen, Jianhui Xie

**Affiliations:** 1 Department of Forensic Medicine, Shanghai Medical College of Fudan University, Shanghai, China; 2 Department of Biochemistry and Molecular Biology, Shanghai Medical College of Fudan University, Shanghai, China

## Abstract

**Aim:**

To systemically select and evaluate short tandem repeats (STRs) on the chromosome 14 and obtain new STR loci as expanded genotyping markers for forensic application.

**Methods:**

STRs on the chromosome 14 were filtered from Tandem Repeats Database and further selected based on their positions on the chromosome, repeat patterns of the core sequences, sequence homology of the flanking regions, and suitability of flanking regions in primer design. The STR locus with the highest heterozygosity and polymorphism information content (PIC) was selected for further analysis of genetic polymorphism, forensic parameters, and the core sequence.

**Results:**

Among 26 STR loci selected as candidates, D14S739 had the highest heterozygosity (0.8691) and PIC (0.8432), and showed no deviation from the Hardy-Weinberg equilibrium. 14 alleles were observed, ranging in size from 21 to 34 tetranucleotide units in the core region of (GATA)_9-18_ (GACA)_7-12_ GACG (GACA)_2_ GATA. Paternity testing showed no mutations.

**Conclusion:**

D14S739 is a highly informative STR locus and could be a suitable genetic marker for forensic applications in the Han Chinese population.

Short tandem repeats (STRs) comprise the repeat units of 2 base pairs (bp) to 7 bp in length (1). Due to a high degree of length polymorphism as a result of variation in the number of repeat units and a short size of amplification products, they have become the most popular genetic markers for the identification of individuals and paternity testing (2). However, only a small number of STRs with high degree of length polymorphism is suitable for use as genotyping markers. Multiplex assays commonly include non-coding tetranucleotide and pentanucleotide repeats, which enables high combined power of discrimination (CPD) and combined power of exclusion (CPE) in a single test. Currently, commercial kits, such as PowerPlex^®^ Fusion System (Promega, Madison, WI, USA) and GlobalFiler^®^ Express Kit (Thermofisher Scientific Inc., Waltham, MA, USA) allow simultaneous amplification of more than 20 autosomal STR loci, which simplifies forensic DNA profiling (3,4).

STRs are prone to mutation in meiosis, which might result in a false maternal or paternal exclusion due to gain or loss of repeat units. Therefore, additional genetic information is required to increase the combined paternity index (CPI), which allows the detection of true parental relationships in a pedigree and reduces the chances of false exclusion. Currently, commercially available kits include some STR loci with a low power of discrimination (PD) and low power of exclusion (PE), such as TPOX. Furthermore, STRs included in Combined DNA Index System (CODIS) and European Standard Set (ESS) belong to only 18 of the 22 autosomal chromosomes (5). Therefore, some new multiplex STR typing systems were developed to provide additional information for paternity testing, such as 26plex STR assay (6). However, most STR loci used in the expanded assays, such as D14S1434, also have low PD and PE (7).

The development of six dyes permits a simultaneous detection of more STR loci in a multiplex STR typing system (4). CPD and CPE can be increased if an STR locus with low PD and PE in a multiplex STR typing system is replaced by a new STR locus with high PD and PE from the same chromosome, or if such a locus is added to the multiplex STR typing system. This is especially important for new STR loci with high PD and PE from the chromosomes that are not included in multiplex STR typing systems. The addition of these may help to avoid linkage potential between STR loci. Therefore, it is necessary to systemically select and evaluate new STR loci as genotyping markers for forensic application (8). For this purpose we intended to identify STR loci with high degree of polymorphism on chromosome 14. In fact, no STR locus on chromosome 14 has been included in common multiplex STR typing systems, even the latest PowerPlex^®^ Fusion System and GlobalFiler^®^ Express Kit. Although several STR loci on chromosome 14 have been used as expanded genotyping markers, including D14S1434 and D14S608, the use of these loci has several disadvantages. D14S1434 has been reported to have low PD and PE (9) and while D14S608 has relatively high PD, its allele frequency does not show normal distribution in all tested populations (10-14). D14S608 was also observed to have significant deviation from Hardy-Weinberg equilibrium (HWE) in German population (11).

In this study, STR loci on chromosome 14 were filtered from the Tandem Repeats Database (TRDB) (15) and their core and flanking sequences were further evaluated. D14S739 was shown to be highly polymorphic in a small sample size and was further characterized in the Han Chinese population.

## Materials and methods

### Selection of STR loci

A total of 386 repeats on chromosome 14 were preliminarily filtered from TRDB using the following rules: ‘Pattern Size’ was equal to 4; ‘Copy Number’ was ≥8 and ≤30; ‘the content of GC’ was 20%-55%, ‘%Indels’ was equal to 0, and ‘%Matches’ was ≥90%. A set of 26 STR loci was selected based on the positions on the chromosome, repeat patterns of core sequences, sequence homology of flanking regions, and suitability of flanking regions in primer design.

### Primer design, amplification, and electrophoresis

Primers were designed by using Primer v5.0 (Premier Biosoft Interpairs, Palo Alto, CA, USA). The amplification of STR loci was performed by polymerase chain reaction (PCR) including 2.5 μL 10 × PCR buffer (with MgCl_2_), 2.0 μL deoxynucleotide mixture (2.5 mM), 1.0 μL FAM^TM^-labeled or unlabelled primer set (100 μM, Sangon Biotech., Shanghai, China), 1.0 μL rTaq DNA polymerase (5U/μL), and 1.0 μL sample DNA in a 25 μL final reaction volume. After an initial denaturation at 94°C for 3 minutes, PCR was carried out for 31 cycles under the following conditions: denaturation at 94°C for 30 seconds, annealing at 58°C for 35 seconds, extension at 72°C for 30 seconds, and a final extension at 72°C for 25 minutes. PCR products were separated by agarose gel electrophoresis or capillary electrophoresis in ABI PRISM 3130xL Genetic Analyzer (Thermofisher Scientific Inc.).

### Naming of the alleles and allelic ladder

The pilot investigation of genetic polymorphism was performed with 35 individual DNA samples. The number of alleles of each STR locus was determined and the forensic parameters were evaluated. The PCR products of each allele were cloned in plasmid vectors and sequenced by 3130xL Genetic Analyzer. The alleles were named according to the sequencing results and the recommendations of the DNA Commission of the International Society of Forensic Genetics (ISFG) (16). The alleles were amplified, and then the products were diluted, mixed together, analyzed, and balanced to produce the allelic ladder (17). Panel and bin files for GeneMapper ID software v3.2 were programmed by using fixed size of allelic ladder.

### Population investigation and data analysis

The bloodstains were collected from 511 unrelated individuals after informed consent had been obtained and the DNA samples were prepared by 10% Chelex-100 solution (Bio-Rad Laboratories, Hercules, CA, USA) and proteinase K (18). The allelic ladder, panel, and bins were updated when new alleles were observed. The values for allele frequencies, observed heterozygosity (Ho), expected heterozygosity (He), polymorphism information content (PIC), PD, PE were calculated, and the exact test of HWE was performed using the PowerStats v1.2 software (19) and PowerMarker software v3.25 (20). The study was approved by the ethics committee of Shanghai Medical College, Fudan University.

## Results

### Selection of STR loci on chromosome 14

**Figure 1:**
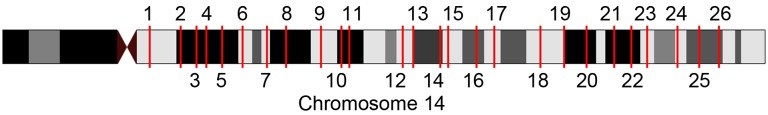
The location of the 26 investigated loci on the chromosome 14.

From a total of 27 552 loci in TRDB, we obtained 386 STR loci. The sequence homology of flanking regions was evaluated by the Blat tool (*http://genome.ucsc.edu/cgi-bin/hgBlat*) and the suitability of flanking regions in primer design was assessed by Oligo v. 7.0 software (Molecular Biology Insights, West Cascade, CO, USA). A set of 26 STR loci with a spacing of about 3 Mb from each other was selected for further investigation (Figure 1 and Table 1).

**Table 1:** Position, repeat pattern, primers, and alleles of the investigated 26 short tandem loci

No.	Position	Pattern	Primer		Alleles
1	chr14:21095068-21095100	TGTT	F: CAGCCCGTCACCTACAGAAGT	R: GCGACATAGACAGACCATTATCAG	2
2	chr14:24928870-24928908	ATAG	F: CAGGGCAGTGAATGAGAAATG	R: CCTCTGAGTGAGTAGGAAATGGAA	5
3	chr14:27285629-27285674	TCTA	F: ATGTGGTACAATATGGTCGTTTGAG	R: CTGTTCTTAAAGGTGGGATACAGTAG	6
4	chr14:28516687-28516740	ATAG	F: ATGTGGTACAATATGGTCGTTTGAG	R: CTGTTCTTAAAGGTGGGATACAGTAG	5
5	chr14:31208279-31208336	TGTA	F: CAGTGTCATAAGGTCCTTGATTGG	R: CAAGAGCTTCTGGGTTGCTGAAC	5
6	chr14:33753951-33754021	TATC	F: CTGGCAGAAGACAACTCATTACCT	R: AACTACTACAGGCAGATGTTGAGAAG	1
7	chr14:37388285-37388320	AATG	F: TGAGATTTCATGTTCTTCCCACTG	R: CATATCATGTTCAAATATGGCAAAGA	1
8	chr14:40164671-40164734	ATCT	F: TAAAATATCCCACATAATGCCTACC	R: ACACCAGGACTTGGAAAGGAAT	6
9	chr14:44936745-44936785	ATAC	F: CAGATCTAATTTTGGGTTCACATTCA	R: TGCATTTTGCAGACTTAGTCTTAGC	6
10	chr14:47570279-47570340	GATA	F: GCTCTGTAGTGGGGATACTTGATG	R: AAGAAAACTTTGTTTGCCAGGAG	6
11	chr14:48802193-48802232	ATCT	F: CTAATAGGTAGAATATAAAAGCAAGACTGAC	R: GAATTAGAGAAAGGTTGGGAGAATCAG	1
12	chr14:56412077-56412120	CATA	F: TGGAATTTAACCTAAGAACTGGTAGAGTA	R: CTTACAGCTTATAGATGGTCTACTGTGG	4
13	chr14:58075863-58075899	GATG	F: CGATAAACCATCCCAAATCTGT	R: ACTCAGGGTAGCAAGATTTCCAG	1
14	chr14:61902423-61902460	AATG	F: GTCAGCCTGGGCAATGTAATG	R: AAGAGGCCTTGGCTCCAATAAC	4
15	chr14:63025771-63025808	ATAC	F: TGGTCTAAGTCTTGCTTGTACTGAGG	R: AACCTTTGCTACAGAACTGGCTC	1
16	chr14:66779614-66779650	AAAC	F: TCGTCCTTCCAAAACTAACTTCAC	R: TGGTTTATGGAGCCTTTGGTATC	2
17	chr14:69418540-69418578	TCAT	F: ATGTGGCTTCCCTTTTGGCTAC	R: CTCCCAGAAGTAATGGCTCTAAGTG	1
18	chr14:75613612-75613648	AGAT	F: CAAAGACTGAACAAACTGTCCCAC	R: CAGTTGTTCAAGACTAAACAGCACTGA	6
19	chr14:79328589-79328628	AGAT	F: TACTTAGGGAGACCTTTCTAGTTGGTG	R: TTACTGCTCCCTTAAAGCTATGTGG	5
20	chr14:82266671-82266717	TCTG	F: GAGTGTATTAGGGTTTTCCAGAAGAAC	R: CAGCATACCAGCAGCAGATCTT	9
21	chr14:85940746-85940824	TATC	F: ATGGGAGTGTCATTTAGTTAGATGTATT	R: TGCAAACAAAGTTTGACAAATACG	6
22	chr14:88471185-88471217	ATGA	F: GTACTTCTTCATTTTGCCATTGC	R: GTACTGTAATTTTTATAACTCACTTGTC	1
23	chr14:91004576-91004686	ATGG	F: CACTTTTGAAATGTTTATCCCTCAG	R: AGCCTTTCCCAACTAAGGACATT	6
24	chr14:95025092-95025127	TTCA	F: CCTTCCTGGTCAGGCAACTTATC	R: GCTTCAGGATTGTTGAACCCTTG	6
25	chr14:97938685-97938734	CAAG	F: CTCTTGCACCCCAAAGTATGATG	R: TCAGCTCACGGTGGTAGACAGAC	3
26	chr14:100901981-100902026	CATT	F: TTGGAGATCACGGAAAGAGAAGC	R: TGAAGATCCCCATCTGGACTTG	3

### Pilot investigation of genetic polymorphism

**Figure 2:**
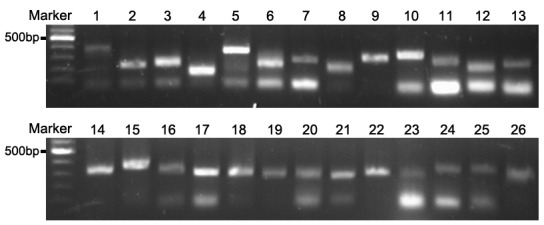
Electrophoretograms of polymerase chain reaction products created by using primer sets for 26 short tandem repeat loci.

The specificity of primer sets for the 26 STR loci was tested by PCR amplification and agarose gel electrophoresis (Table 1 and Figure 2) and further evaluated by capillary electrophoresis. Pilot investigation of genetic polymorphism showed that the locus with the highest heterozygosity, PIC, PD, and PE locus No. 20 with 9 alleles. University of California Santa Cruz (UCSC) Genome browser analysis (http://genome.ucsc.edu) showed that the locus No. 20 had an identical location on chromosome 14 as D14S739. Therefore, D14S739 was further analyzed.

### The population analysis of D14S739

The allelic ladder with 9 alleles of D14S739 was prepared and the genetic diversity of D14S739 in the Han population was investigated. In all tested samples we observed 14 alleles. Insertion-deletion polymorphisms (Indels), which result in microvariants, were not observed. The forensic parameters of D14S739 including allele frequencies, Ho, He, PIC, PD, and PE were calculated and no deviation from HWE was observed (Table 2). Compared with the polymorphism and forensic parameters of CODIS STRs obtained from our laboratory (21), D14S739 was comparable to the FGA locus and superior to other loci.

**Table 2:** The forensic parameters of D14S739

Allele	Frequency	Allele	Frequency
21	0.001	28	0.1377
22	0.0039	29	0.0791
23	0.0117	30	0.0869
24	0.0361	31	0.0693
25	0.1719	32	0.0225
26	0.1543	33	0.0039
27	0.2197	34	0.002
Observed heterozygosity	0.8691		
Expected heterozygosity	0.8588		
Power of discrimination	0.9615		
Probability of exclusion	0.7328		
Polymorphism information content	0.8432		
P*	0.5528		

*Hardy-Weinberg equilibrium exact test.

### The core sequence analysis of D14S739

We next analyzed the sequence of D14S739 in the human genome version 19 (Hg19). In its core region, there are two repeat motifs GTCT and ATCT. However, D14S739 was originally cloned with the oligonucleotide probe of GATA repeats (22). According to the nomenclature for STR alleles, the repeat motifs of D14S739 should be defined as GATA motif and GACA motif (16). To further determine the nucleotide sequences of all 14 alleles, the representative samples containing the alleles of D14S739 were used to amplify the target region and PCR products were cloned into pMD^TM^19-T Simple Vector (Takara, Shiga, Japan) followed by sequencing. Sequencing results showed that the core region of D14S739 was [GATA]_9-18_ [GACA]_7-12_ GACG [GACA]_2_ GATA. The invariant sequence GACG [GACA]_2_ GATA at the 3′-end was considered as repeat units according to the nomenclature recommended by ISFG DNA Commission (23). Thus, the core regions of alleles ranged from 21 to 34 tetranucleotide units with compound GATA/GACA repeat motif.

**Figure 3:**
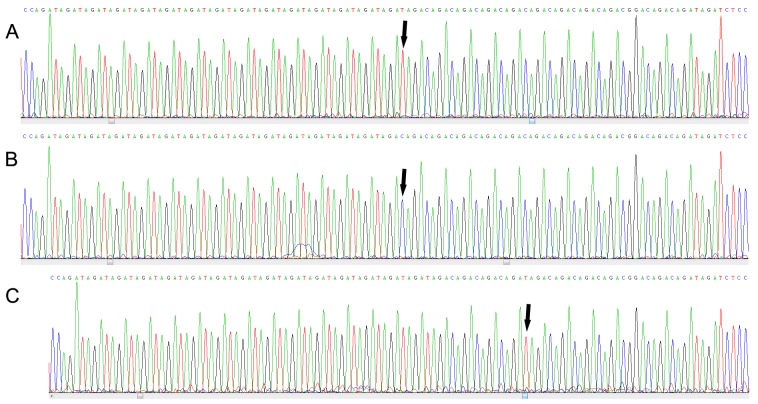
Electrophoretograms of two isometric heterozygotes (**A**) and (**B**) and one microvariant (**C**). Arrow represents variation.

Because of the combination of two repeat motifs in the core region, alleles with the same size had different repeat patterns (Figure 3A and B). The single nucleotide variation in alleles was also observed. The transition of cytosine to thymine in the GACA motif led to the appearance of GATA motif (Figure 3C). Other alleles might have a similar pattern although we did not sequence all the alleles in the population. In fact, the single nucleotide polymorphism in the core region of D14S739 was confirmed by the UCSC Genome Browser Database (*http://genome.ucsc.edu*).

### Detection of D14S739 in paternity testing

**Figure 4:**
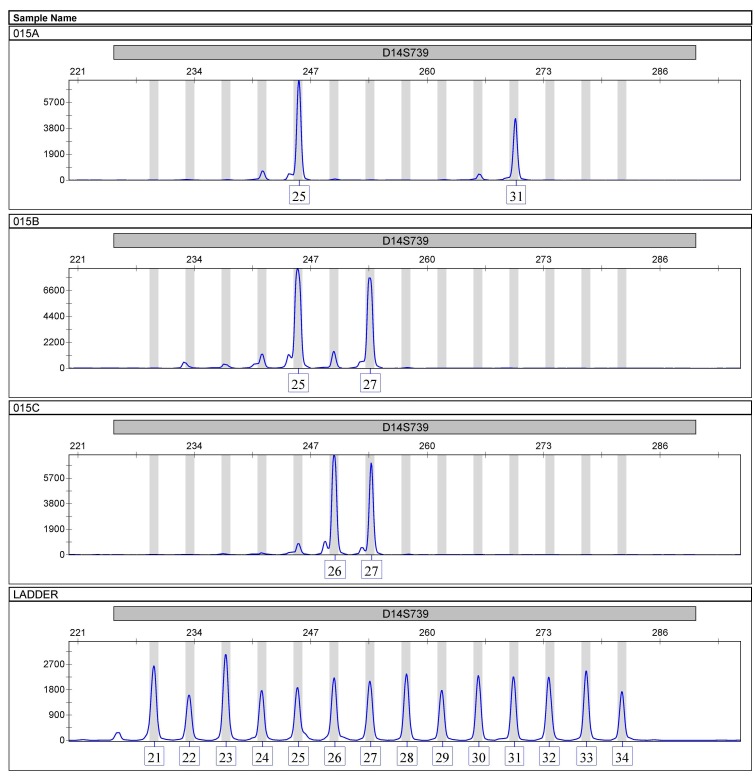
Electrophoretograms of D14S739 in a trio paternity test. The serial numbers 015A, 015B, and 015C represent father, son, and mother, respectively.

The allelic ladder with 14 alleles of D14S739 was prepared and the performance of D14S739 in paternity testing was investigated in 200 trio paternity tests using PowerPlex^®^ 21 System (Promega, Madison, WI, USA). The transmission of alleles from parents to their offspring conformed to Mendelian laws and no mutation was observed. The representative genotypes of one trio paternity test together with the allelic ladder are shown in Figure 4.

## Discussion

In this study, we performed a comprehensive screening of STR loci on chromosome 14 and identified D14S739 as a highly polymorphic STR locus in the Han Chinese population. Generally, the degree of polymorphism for a genetic locus can be measured by two distinct parameters – heterozygosity and PIC (24). Our results showed that D14S739 had higher heterozygosity (0.8691) and PIC (0.8432) in the Han Chinese population than D14S1434 (0.682 and 0.645, respectively) (9) and D14S608 (0.8110 and 0.8399, respectively) (14). Similarly, D14S739 had higher PD (0.9615) and PE (0.7328) than D14S1434 (0.863 and 0.378, respectively) (9) and D14S608 (0.9504 and 0.6659, respectively) (14). Therefore, the inclusion of D14S739 in multiplex STR typing systems could help to achieve high CPD and CPE.

The addition of independent STR loci with high degree of polymorphism in multiplex STR typing systems could minimize adventitious matches in forensic casework. However, not all STR loci are suitable for the forensic purposes, so thorough evaluation is needed to filter out unsuitable ones (8). In this study, 26 STR loci with a spacing of 3 Mb on the chromosome were used as candidates. It is possible that we missed some of the suitable loci. In fact, it is difficult to evaluate a large number of STRs by manual screening. Therefore, a primary alignment using results from the whole genome sequencing against the reference genome could provide an overall view of the variation of all STR loci in a population, which can decrease the chance of missing polymorphic STR loci during the screening.

D14S739, also known as GATA65G10, was first cloned with the oligonucleotide probe of GATA repeats and subsequently used for the construction of human genetic maps (22,25). The transition of cytosine to thymine creates GATA motif in the repetitive GACA region having as a consequence that alleles with the same size have different DNA sequences. The sequence variants make it difficult to accurately determine the DNA sequence of alleles with the same size. In these cases sequencing of a large number of alleles should be performed. The sequence polymorphism in the repeat motif was also observed in other STR loci, such as vWA (26). The internal allele variation might not be an important consideration in forensic casework since STR variation is primarily size-based and alleles of several STR loci with the same size, such as D21S11 and FGA, contain variable repeat blocks in the core region (26). In this study, the alleles of D14S739 were named based on the size of the core region. The size-based variation of D14S739 leads to a high degree of polymorphism, and therefore has enough discriminating power for forensic purposes. Besides the fragment length, the sequence variation of D14S739 can provide additional information for the application of next-generation sequencing in forensic practice.

During meiosis an STR locus might lose or gain one or more repeat units, which affects the interpretation of paternity testing results. Previous studies showed the highest mutation rate for FGA and D21S11, which can be derived from the relatively large number of repeat units (27). However, FGA and D21S11 alleles with incomplete repeat units were widely observed (28). We did not observe incomplete repeat units at D14S739 locus, although D14S739 has 21-34 repeat units. We also did not observe mutation of D14S739 in 200 trio paternity tests. Therefore, D14S739 might have a relatively low mutation rate during meiosis. Since this study had a relatively small sample size, studies with larger sample sizes are needed to further determine the mutation rate of D14S739.

## References

[1] Subramanian S, Mishra RK, Singh L (2003). Genome-wide analysis of microsatellite repeats in humans: their abundance and density in specific genomic regions.. Genome Biol.

[2] Weber JL, Broman KW (2001). Genotyping for human whole-genome scans: past, present, and future.. Adv Genet.

[3] Oostdik K, Lenz K, Nye J, Schelling K, Yet D, Bruski S (2014). Developmental validation of the PowerPlex((R)) Fusion System for analysis of casework and reference samples: A 24-locus multiplex for new database standards.. Forensic Sci Int Genet.

[4] Flores S, Sun J, King J, Budowle B (2014). Internal validation of the GlobalFiler Express PCR Amplification Kit for the direct amplification of reference DNA samples on a high-throughput automated workflow.. Forensic Sci Int Genet.

[5] Guo F, Shen H, Tian H, Jin P, Jiang X (2014). Development of a 24-locus multiplex system to incorporate the core loci in the Combined DNA Index System (CODIS) and the European Standard Set (ESS).. Forensic Sci Int Genet.

[6] Hill CR, Butler JM, Vallone PM (2009). A 26plex autosomal STR assay to aid human identity testing.. J Forensic Sci.

[7] Yuan L, Ge J, Lu D, Yang X (2012). Population data of 21 non-CODIS STR loci in Han population of northern China.. Int J Legal Med.

[8] Hares DR (2012). Expanding the CODIS core loci in the United States.. Forensic Sci Int Genet.

[9] Bai R, Shi M, Yu X, Lv J, Tu Y (2007). Allele frequencies for six miniSTR loci of two ethnic populations in China.. Forensic Sci Int.

[10] Choi M, Kim JH, Lee DH, Lee SH, Rho HM (2000). Frequency data on four tetrameric STR loci D18S1270, D14S608, D16S3253 and D21S1437 in a Korean population.. Int J Legal Med.

[11] Becker D, Vogelsang D, Brabetz W (2007). Population data on the seven short tandem repeat loci D4S2366, D6S474, D14S608, D19S246, D20S480, D21S226 and D22S689 in a German population.. Int J Legal Med.

[12] Asamura H, Ota M, Fukushima H (2008). Population data on 10 non-CODIS STR loci in Japanese population using a newly developed multiplex PCR system.. J Forensic Leg Med.

[13] Hwa HL, Chang YY, Lee JC, Yin HY, Tseng LH, Su YN (2011). Fourteen non-CODIS autosomal short tandem repeat loci multiplex data from Taiwanese.. Int J Legal Med.

[14] Zhang S, Tian H, Wu J, Zhao S, Li C (2013). A new multiplex assay of 17 autosomal STRs and Amelogenin for forensic application.. PLoS ONE.

[15] Gelfand Y, Rodriguez A, Benson G (2007). TRDB – the Tandem Repeats Database.. Nucleic Acids Res.

[16] Lincoln PJ (1997). DNA recommendations – further report of the DNA Commission of the ISFH regarding the use of short tandem repeat systems.. Forensic Sci Int.

[17] Griffiths RA, Barber MD, Johnson PE, Gillbard SM, Haywood MD, Smith CD (1998). New reference allelic ladders to improve allelic designation in a multiplex STR system.. Int J Legal Med.

[18] Butler JM (2009). DNA extraction from forensic samples using chelex. Cold Spring Harb Protoc.

[19] PowerStats Version 1.2, Promega Corporation Website. Available from: http://www.promega.com/geneticidtools/powerstats/. Accessed: May 15, 2001.

[20] Liu K, Muse SV (2005). PowerMarker: an integrated analysis environment for genetic marker analysis.. Bioinformatics.

[21] Xie J, Shao C, Zhou Y, Zhu W, Xu H, Liu Z (2014). Genetic distribution on 20 STR loci from the Han population in Shanghai, China.. Forensic Sci Int Genet.

[22] Sheffield VC, Weber JL, Buetow KH, Murray JC, Even DA, Wiles K (1995). A collection of tri- and tetranucleotide repeat markers used to generate high quality, high resolution human genome-wide linkage maps.. Hum Mol Genet.

[23] Gusmao L, Butler JM, Carracedo A, Gill P, Kayser M, Mayr WR (2006). DNA Commission of the International Society of Forensic Genetics (ISFG): an update of the recommendations on the use of Y-STRs in forensic analysis.. Forensic Sci Int.

[24] Shete S, Tiwari H, Elston RC (2000). On estimating the heterozygosity and polymorphism information content value.. Theor Popul Biol.

[25] Kong A, Gudbjartsson DF, Sainz J, Jonsdottir GM, Gudjonsson SA, Richardsson B (2002). A high-resolution recombination map of the human genome.. Nat Genet.

[26] Lazaruk K, Wallin J, Holt C, Nguyen T, Walsh PS (2001). Sequence variation in humans and other primates at six short tandem repeat loci used in forensic identity testing.. Forensic Sci Int.

[27] Yan J, Liu Y, Tang H, Zhang Q, Huo Z, Hu S (2006). Mutations at 17 STR loci in Chinese population.. Forensic Sci Int.

[28] Ruitberg CM, Reeder DJ, Butler JM (2001). STRBase: a short tandem repeat DNA database for the human identity testing community.. Nucleic Acids Res.

